# Medicinal Effect of Nutraceutical Fruits for the Cognition and Brain Health

**DOI:** 10.1155/2016/3109254

**Published:** 2016-02-04

**Authors:** Raj K. Keservani, Anil K. Sharma, Rajesh K. Kesharwani

**Affiliations:** ^1^School of Pharmaceutical Sciences, Rajiv Gandhi Proudyogiki Vishwavidyalaya, Bhopal 462036, India; ^2^Department of Pharmaceutics, Delhi Institute of Pharmaceutical Sciences and Research, New Delhi 110017, India; ^3^Department of Biotechnology, NIET, NIMS University, Shobha Nagar, Jaipur, Rajasthan 303121, India

## Abstract

The recent era is witnessing evaluation of medicinal and nutritional value of fruits and fruit juices for the management and prevention of brain diseases like headache stress, anxiety, hypertension, and Alzheimer's and Parkinson's diseases by the scientists and researchers worldwide. Fruits possess various chemicals such as antioxidants and polyphenols, which reduce and balance the effect of hormone in brain responsible for brain disease. Natural remedy is cheap, easily available, nontoxic, and easy to prepare and provides good mental health as compared to other remedies. The main objective of this review is to acknowledge medicinal benefits of fruits for the cognition and management of brain disease.

## 1. Introduction

The potential of food as prophylactic and therapeutic agent versus diseases has now begun to be acknowledged. The fascinating facts have been revealed about effect of dietary factors on certain molecular systems and mechanisms that take care of mental activity in recent years. A diet that is high in omega-3 fatty acids is earning accolades for reinforcing cognitive activity in humans [1] and upregulating genes that are vital for sustaining synaptic activity and agility in rodents [2]. Eventually, diets that are rich in saturated fat are turning culprits for lowering molecular scaffolds that help cognitive function and elevating the danger of neurological dysfunction in humans [3] as well as animals [4]. Thus, a combination of nutrition and exercise is recommended to undo these probable ill health outcomes. This is further corroborated by information available in the literature [5].

Nutraceutical is hybrid of nutrition and pharmaceutical and was introduced in 1989 by Stephen L. DeFelice, founder and chairperson of the Foundation of Innovation Medicine, and defined as “Food, or parts of food, that provide medical or health benefits, including the prevention and treatment of disease” [6, 7]. Functional foods, according to their generally accepted definition, are “any food or food ingredient that may provide a health benefit beyond the traditional nutrients it contains” [8]. Plenty of studies are about fruitful influences of nutraceuticals like antioxidant, mushrooms, vitamins, essential amino acids, phytochemicals, and polyunsaturated fatty acids in pediatric foods upon the budding immune response [7, 9].

There are numerous evidences regarding effect of general physical condition on children's cognitive ability and school performance [10, 11]. The activity of brain is definitely based on appropriate nutrition, and short-term alterations in the quantity and makeup of nutrient intake in healthy individuals affect actions of cognitive processes. It has been observed that consuming breakfast leads to a number of affirmative influences on the cognitive activity of well-fed children [11, 12]. The elevated hormone content and enzyme functions signal towards sensitivity of brain for stress [13]. The high stress circumstances may trigger depression and in adverse manner alter behavioral, learning, and biochemical activities. A mood disorder such as major depression is probable life endangering disease. A sizeable population of patients with depression is reckoned to be treatment resistant even after advancement of pharmacotherapy [14]. The antidepressant drugs suffer from drawbacks such as postcompliance side effects or absence of required effect [15, 16]. These findings have ignited the minds of researchers to pursue hunt for natural remedies for improved beneficial effects with no or lesser toxic consequences. The dementia is often manifested by Alzheimer's disease. It causes severe suffering for the patients, in the form of progressive behavioral and neurological changes that include functional impairment, loss of independency, emotional problems, and behavioral disturbances [17].

A high risk of many mental disorders has been observed, such as attention-deficit disorder, dyslexia, dementia, depression, bipolar disorder, and schizophrenia when humans are omega-3 fatty acids deficient [18–22]. Since the human body is not able to synthesize omega-3 fatty acid docosahexaenoic acid (DHA) that is an important part of neuronal membranes, we have to depend on DHA from dietary sources. The mechanisms underlying DHA's action on brain plasticity and cognition have begun to be explained in recent times. The enhancement of hippocampal brain-derived neurotrophic factor (BDNF) content and increased cognitive process in rodent models of brain trauma are observed during DHA dietary supplementation [23].

## 2. Nutraceutical Fruits for Cognition and Brain Health

**Figure 1:**
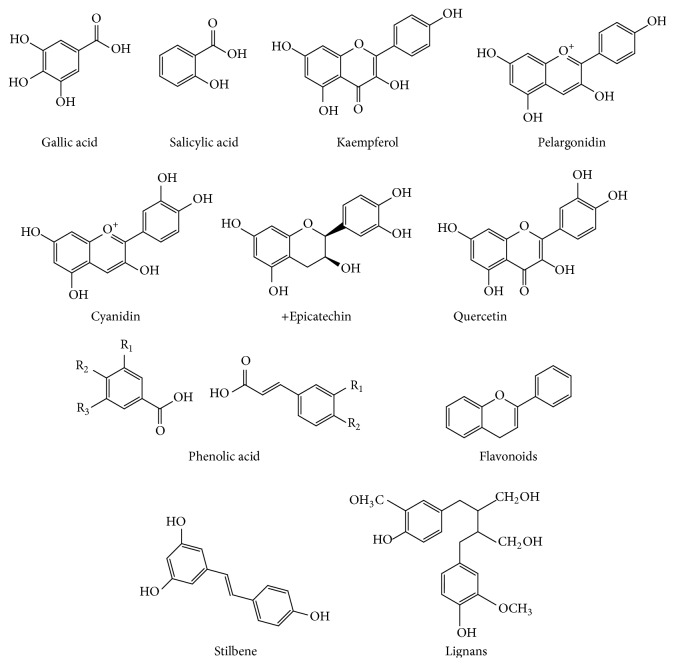
Structure of polyphenols found in fruits.

Fruits and fruit juice were used for cognition and brain health. Keservani and coworkers reported the use of various fruits for brain health because of medicinal value of fruits [24, 25]. Keservani and Sharma reported the role of vitamin C and polyphenols found in citrus fruits and blueberries in mental performance [26]. Detailed description of blackberries, blueberries, strawberries, raspberries, cherries, oranges, plums, prunes, red grapes, and pomegranates fruits is mentioned in the following sections. The numerous phytochemicals occurring in these fruits are listed in Table 1 and their structures are depicted by Figure 1.

### 2.1. Blackberries

**Figure 2:**
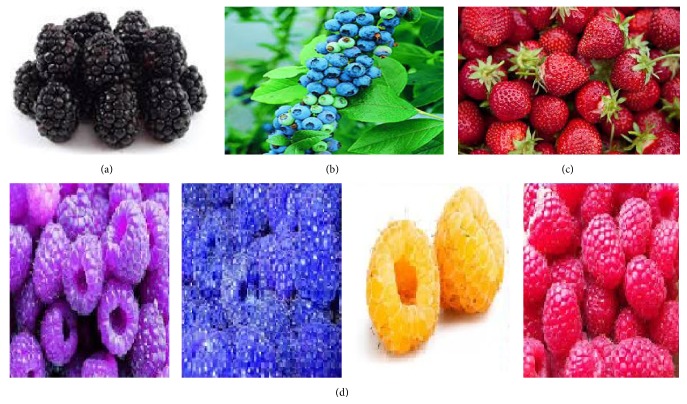
Structure of nutraceutical fruits for brain health: (a) blackberries, (b) blueberries, (c) strawberries, and (d) raspberries (purple, blue, yellow, and red).

Several species in the* Rubus* genus of Rosaceae family, hybrids of these species in the* Rubus* subgenus, and hybrids between the* Rubus* and* Idaeobatus* subgenera provide blackberry (Figure 2(a)) which is an edible fruit [27]. Blackberries are noteworthy, as these possess rich nutritional amounts of dietary fiber, vitamin C, and vitamin K. Blackberries contain soluble as well as insoluble fiber. The half of the daily recommended dose of vitamin C may be provided by 1 cup of blackberries (144 g) which contain a mean of 7.6 g of fiber.

By virtue of having high polyphenol and anthocyanin content (often collectively known as phytochemicals) red and dark colored berries offer health benefits which are documented too. Nevertheless, researchers around the globe persist to express interest in this region. There has been initial research with focus on the phytochemical makeup of new and much exotic fruits and their comparison with known relatives, berries. A review written by Dr. Miller and Shukitt-Hale of the United States Department of Agriculture (USDA) human nutrition center at Tufts University proposes that intake of blueberries, strawberries, blackberries, and other berry fruits has a valuable effect on the brain and could assist in checking age-linked memory loss and other alterations. The group pleads that the berries possess neuroactive phytochemicals that act as antioxidants as well as anti-inflammatory agents [28].

Berries are famous for their accrual of antioxidant parts (mostly polyphenols, carotenoids, and vitamin C) and for being the fruits offering maximum antioxidant capacity in usually consumed foods [29]. Total polyphenol concentration may also differ at large between berry species and varieties and under different growing environments. Often black currant, raspberry, and strawberry contain total polyphenol amounts in the range of 300–1000 mg/100 g [30]. Besides, the amounts of these antioxidant substances may be hugely affected by postproduction handling and processing [31]; therefore, their content must be authenticated in any product. Recently the role of polyphenol contents in berry as neuroprotective has been documented [28]. The berry ingredients are suggested to guard against damage caused by reactive oxygen species (ROS), which are identified to have role in the progress of neurological conditions like Alzheimer's disease [32]. An improvement in indices of neuronal process in aged rats was observed upon dietary supplementation with blueberry, cranberry, or black currant fruit for eight weeks [33].

### 2.2. Blueberries

Blueberries (Figure 2(b)) are perennial flowering plants having indigo colored berries of the section Cyanococcus in the genus* Vaccinium* (a genus that covers cranberries, bilberries, and gooseberries). Species in the section Cyanococcus are the usual fruits advertised as blueberries and are inhabitant to North America (commercially grown high-bush blueberries were not launched in Europe until the 1930s) [34].

In general, water (84%), carbohydrates (9.7%), proteins (0.6%), and fat (0.4%) are principal ingredients of a fresh blueberry. A serving of fresh blueberries (100 g) is approximated to provide energy amounting to about 192 kJ. Blueberries offer a nice supply of dietary fiber that makes 3–3.5% of its weight. In addition to the taste, the chief attention in this fruit is because of the fair vitamin C content, as 100 g of blueberries offers, on average, 10 mg of ascorbic acid, which equals 1/3 of the daily recommended intake [35, 36].

More recently, studies addressing the effects of other flavonoid subgroups on human cognition have indicated that both blueberry anthocyanins and cocoa flavanols promote positive effects on cognitive outcomes, especially in aged populations. For example, cocoa flavanols (520–994 mg of total cocoa flavanols) have been shown to enhance cognitive and visual function in healthy young volunteers within 2 h of intake, specifically in highly effortful/demanding tasks [37, 38]. On the other hand, long-term supplementation (3 months) with blueberry juice in grown-up adults with slight cognitive destruction yielded working memory improvements and further reduced depressive symptoms [39]. An improved short-term cognitive action with high flavonoid fruit juices, including blueberry juice, was observed in a few trials in geriatric population (≤15 participants) [39, 40]. Corroborating these results, berries are in particular rich in a subclass of flavonoids known as anthocyanidins, which can cross the blood brain barrier and concentrate in regions of learning and memory (e.g., hippocampus) [41].

### 2.3. Strawberries

The garden strawberry (Figure 2(c)) (or simply strawberry;* Fragaria ananassa*) is a broadly grown crossbreed species of the genus* Fragaria* (altogether called as the strawberries) [42]. The garden strawberry was at first reared in Brittany, France, in the 1750s through a cross of* Fragaria virginiana* from eastern North America and* Fragaria chiloensis*, which was fetched from Chile by Amédée-François Frézier in 1714. As evident from epidemiological studies the lower incidences of hypertension, inflammation, cancer, and death from cardiovascular diseases are reported with strawberry consumption [43].

In a study the investigators tested whether long-term (from 6–15 months of age; F344 rats) consumption of a control diet (AIN-93) or a diet supplemented with a strawberry or spinach extract that had been termed as being rich in antioxidant potential by the oxygen radical absorbance capacity assay [44–46] or vitamin E would avert the effects of aging. The diets high in strawberries can also possess the ability to avail benefits to the aging brain, which is exhibited by initial animal studies [47]. Afterwards a number of studies have evaluated the influences of antioxidant and polyphenol high materials on cognitive process. The proofs recommend that these components may certainly affect cognition acutely (when given in high doses) as well as upon long-term intake in both animals and humans [48].

### 2.4. Raspberries

The raspberry (Figure 2(d)) is the edible fruit of a large number of plant species in the genus* Rubus* of the rose family, majority of which are in the subgenus* Idaeobatus*; the nomenclature is also applicable to such plants themselves. The hybrids between* R. idaeus and R. strigosus* are commercially employed to get red raspberry nowadays. Purple raspberries have been cultivated by horticultural cross of red and black raspberries and have been present in the wild (for instance, in Vermont) where the American red and the black raspberries both grow in nature. Blue raspberry is a vernacular name prevailing in Prince Edward Country, Ontario, Canada. Red raspberries have also been hybridized with different species in other subgenera of the genus* Rubus*, yielding numerous hybrids; the first of them was the loganberry. Subsequently remarkable crossbreeds encompass boysenberry (a multigeneration hybrid) and tayberry. Further cross between the familiar grown red raspberries and a few Asiatic species of* Rubus* has also been attempted. Raspberries possess anthocyanin pigments, ellagic acid (from ellagotannins, e.g., the polyphenol ellagitannin), quercetin, gallic acid, cyanidins, pelargonidins, catechins, kaempferol, and salicylic acid [49, 50]. Yellow raspberries and others having yellow colored fruits are poor in anthocyanins [49]. The yellow as well as red raspberries possess carotenoids, often lutein esters, but these are covered by anthocyanins in red raspberries [51].

The berry ingredients are suggested to guard against damage caused by ROS, which are known to be involved in the progress of neurological diseases like Alzheimer's disease [28, 32]. The inferences from in vitro investigations have also been exploited to reinforce the probability that polyphenols present in berries may positively remodel amyloid-beta aggregation [52] in vitro, a process that eventually leads to brain damage in Alzheimer's disease. Blueberry extracts defended against damage caused by inflammation of microglial cells via decrease in inflammatory mediators [53]. In a relevant investigation with concord grape juice, which has several polyphenol components, that also occurs in berries, supplementation refined memory behavior in older adults with predescribed slight cognitive problems, which was assisted by studies on brain activity employing functional magnetic resonance imaging [54].

### 2.5. Cherries

**Figure 3:**
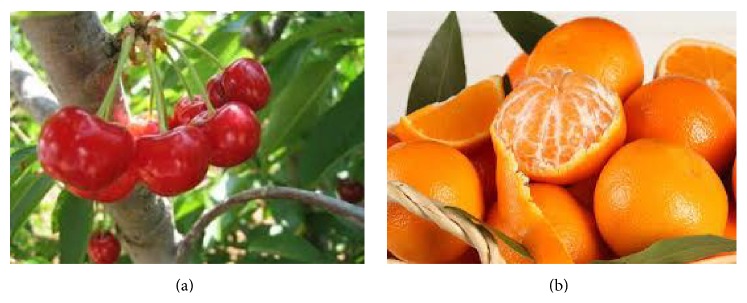
Structure of nutraceutical fruits for brain health: (a) cherries and (b) oranges.

The cherry (Figure 3(a)) fruits possessing market value are often procured from restricted types of sources like varieties of the sweet cherry,* Prunus avium*. Several cherries fall under subgenus* Cerasus*, which is demarcated by bearing the flowers in tiny corymbs of many in groups (neither alone nor in racemes) and by possessing even fruit having only a faint furrow either with side or without groove [55]. Majority of edible cherries are obtained either from* Prunus avium*, the sweet cherry (also known as wild cherry), or from* Prunus cerasus*, the bitter cherry.

There continues to be heightened attention pertaining to application of fruits and vegetables (functional foods) and, in particular, cherries (and other dark fruits) to treat a range of ailments ranging from sleep disorders [56], arthritis [57], muscle damage, and soreness [58] improved cognitive function in Alzheimer's mouse models [59]. The varieties of biological samples have been evaluated for antioxidant activity of cherry extracts. Further, Tsuda et al. [60, 61] exhibited that cyanidin 3-glucoside was found to have best antioxidant activity among the anthocyanins evaluated. The neuronal PC 12 cells subjected to oxidative stress validated antioxidant activity of cherry phenolics [62].

### 2.6. Oranges

Oranges (Figure 3(b)) are the most famous citrus harvests and make up 75% of all cultivated citrus fruits. The “Queen” orange [*Citrus sinensis* (L.) Osb] is cultivated at large scale in Iran. This breed of orange is midseason, of red color, rich in dissolved solids, high in flavor, and seedless to some extent and bears fruits on the tree in a robust manner. The tree is strong, extremely prolific, and immune towards cold [63].

Each 100 g plateful of orange pulp offers approximately 64% of vitamin C daily requirements. Several extra vital nutrients are found in low concentrations. A variety of phytochemicals, such as carotenoids (beta-carotene, lutein and beta-cryptoxanthin), flavonoids (e.g., naringenin) [64], and several volatile organic substances giving orange aroma, like aldehydes, esters, terpenes, alcohols, and ketones, are present in orange [65].

The healthy older adults may be benefitted in terms of cognitive function if exclusively orange juice high in flavanone is consumed for 8 weeks. There is need to elucidate mechanisms of decline in cognitive processes upon aging and the influence of foods and drinks high in flavanone [66]. The spinach, orange juice, and yeast are good sources of folate or folic acid. Subsequent to absorption of vitamin B in the intestine, there is production of a number of forms of folate in the liver. Several physiological abnormalities during development and maturity often resulted due to lack of dietary folate intake [67]. Adequate brain functioning warrants optimum amounts of folate as folate deficiency may result in neurological disorders, like depression [68] and cognitive impairment. The age-linked fall in cognitive activity could be checked by intake of folic acid as adjuvant for 3 years as apparent from inferences of latest randomized clinical trial [69].

### 2.7. Plums

**Figure 4:**
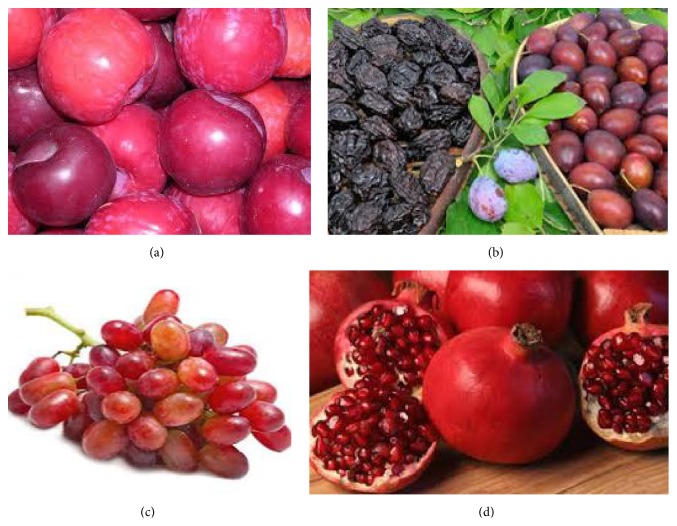
Structure of nutraceutical fruits for brain health: (a) plumes, (b) prunes, (c) red grapes, and (d) pomegranate.

The plum (Figure 4(a)) comes in the subgenus* Prunus* of the genus* Prunus*. The subgenus is differentiated from other subgenera (such as peaches, cherries, and bird cherries) in the shoots with a terminal bud and lonely side buds (not grouped), the flowers in bunch of 1–5 together on short stems, and the fruit bearing a furrow along one side and a flat stone (or pit) [70].

Besides berries, the plums are also high at flavonoid content, in particular in anthocyanins, and could be a decent supply of antioxidants from nature [71]. A control over cognitive decline was observed in aged rats upon consuming plum juice [72]. Kuo et al. investigated effect of a cholesterol rich diet fortified with polyphenols from Oriental plums (Prunus salicina) on cognitive function. The researchers reached conclusion that the cholesterol rich diet per se resulted in outstanding cognitive decline, which was escorted by a superbly elevated mRNA expression of Cyp46, BACE1, A*β*, and 24-hydroxycholesterol within the brain cortex and hippocampus. Nevertheless, all of these factors were nonsignificantly increased in the high oriental plum group (HOP) against control group. Thus, addition of polyphenol-fortified Oriental plum to a cholesterol rich diet may alleviate few of the manifestations of neurodegenerative disorders [73]. The fruits like blueberries, kiwis, plums, and apples have chlorogenic acid. The resveratrol, principal stilbene, may be occurring in the cis or trans configurations, either glucosylated (piceid) or in lesser amounts as the parent molecule of a class of polymers like viniferine, pallidol, or ampelopsin A. The dietary sources of resveratrol are grapes, wine, and peanuts [74].

### 2.8. Prunes

A prune (Figure 4(b)) is a dried plum of any cultivar, mostly* Prunus domestica* or European Plum. The use of the term for fresh fruit is obsolete except when applied to varieties grown for drying [75]. The presence of appreciable concentrations of vital nutrients such as carbohydrates, vitamins, and minerals renders prunes as healthy food. The medicinal significance of prunes and their products is also reported [76]. Prunes provide main nutrients, embracing carbohydrates, many amino acids, vitamin A, vitamin B-complex, vitamin K, potassium, calcium, magnesium, zinc, copper, manganese, selenium, boron, and dietary fibers. The soluble fraction (80%) having pectin, hemicellulose, cellulose, and lignins form prunes fiber. The total dietary fibers are enhanced upon drying [77].

The anxiolytic effect of chlorogenic acid, in a dose of 20 mg/Kg, has been demonstrated in mouse models of anxiety, which may be via the activation of benzodiazepine receptors [78]. The pathogenesis of anxiety conditions has recently been found to implicate role of oxidative stress in brain. The presence of chlorogenic acid and ability to enhance antioxidant protection make prunes promising in anxiety disorders [79].

### 2.9. Red Grapes

The investigations have revealed that manifestations of cancer, heart disease, degenerative nerve disease, viral infections, and Alzheimer's disease are inhibited by phytochemicals present in grapes [like resveratrol (a polyphenol)] [80]. A common activity of resveratrol is responsible for defense of genome via antioxidant activity [81]. In laboratory research using mice, resveratrol is transcriptionally superimposed with the fruitful outcomes of calorie reduction in heart, skeletal muscle, and brain. The dietary measures block gene expression related to heart and skeletal muscle aging and check age-linked heart failure [82].

Globally grape (Figure 4(c)) is one of the most generally eaten fruits. It possesses a number of biological activities, owing to its high polyphenol components, and majority of them are present in its seeds (60–70%) and skin (30%). Nevertheless, huge amounts of grape seed wastes resulted annually by the food processing industry, wine, juice, and so forth [83]. Polyphenols from grape seeds may check oxidative destruction to cellular DNA in vitro [84, 85]. Guo et al. [86] investigated the probable shielding outcomes of grape seed oligomer and polymer procyanidin fractions against ethanol-stimulated toxicity. The findings showed that ethanol might trigger region-selective oxidative DNA loss in the cerebellum and hippocampus being more susceptible; however, consumption of grape seed procyanidins or other natural antioxidants could safeguard the brain against ethanol-induced genotoxicity.

### 2.10. Pomegranates

The Carthage where the finest pomegranates (Figure 4(d)) were recognized to grow was assigned genus* Punica* (its roman name) for pomegranate. The French call pomegranate as grenade whereas the Spanish utter it granada, and verbatim it means seeded (granatus) apple (pomum). The pomegranate (*Punica granatum*) is a deciduous shrub or stout tree, with height of 5–8 m (16 and 26 ft) [87]. The edible seeds of pomegranate are a brilliant source of dietary fiber (20% DV). The nutritional benefits offered by the seed fiber and micronutrients are wasted by consumers who opt to discard the seeds. The ingredients such as punicic acid (65.3%), palmitic acid (4.8%), stearic acid (2.3%), oleic acid (6.3%), and linoleic acid (6.6%) are contained in pomegranate seed oil [88]. The most common phytochemicals in pomegranate juice are polyphenols, encompassing the hydrolyzable tannins known as ellagitannins produced when ellagic acid and/or gallic acid attach with a carbohydrate to yield pomegranate ellagitannins, also called punicalagins [89].

The neuroprotective features of pomegranate polyphenols were assessed in an animal model of Alzheimer's disease. Transgenic mice with Alzheimer's mimicking pathogenesis treated with pomegranate juice showed 50-percent fewer deposition of soluble amyloid-beta and few hippocampal amyloid accumulation than mice consuming sugar water, proposing that pomegranate juice can be neuroprotective. Animals too showed better learning of water maze accomplishments and swam faster than control animals [90]. The pregnant mice were provided pomegranate juice in drinking water disclosing that neonatal offspring being exposed to experimentally stimulated hypoxic-ischemic (HI) brain injury had superbly lesser degree of brain tissue damage (64% decrease) and outstandingly reduced hippocampal caspase-3 activity (84% decrease) in comparison with neonates having experimentally induced HI brain injury from dams who were given a control drink [91, 92]. These findings advise that pomegranate juice possesses an antioxidant-governed neuroprotective activity transferred from mother to neonate.

The inhibition of serum angiotensin converting enzyme (ACE) and reduction in systolic blood pressure in hypertensive patients were observed by intake of pomegranate juice as revealed by a small clinical trial. Ten hypertensive individuals (aged 62–77; seven men and three women) were provided 50 mL/day pomegranate juice having 1.5 mmol total polyphenols for two weeks. Two of 7 men patients were diabetic too and two were hyperlipidemic as well. Seven of 10 subjects (70%) perceived a 36-percent mean reduction in serum ACE function and a tiny, but superb, 5-percent lowering of systolic blood pressure [93]. The dietary intake of antioxidants alleviates the danger of Alzheimer's disease as evident from numerous studies. Yet, more experiments are required to devise firm conclusions on the prophylactic effect of antioxidants [94]. Naveen et al. studied antidepression influence of polyphenols and omega-3 fatty acid from pomegranate peel and flax seed in mice subjected to chronic slight stress and summarized that the polyphenols from flax seed having omega-3 fatty acids were able to decrease all the chronic mild stress (CMS) outcomes examined in comparison with polyphenols from pomegranate peel [95]. Subash and colleagues investigated that pomegranate from Oman decreases the brain oxidative loss in transgenic mouse model of Alzheimer's disease (AD) and proposed that the therapeutic caliber of 4% pomegranate in the treatment of AD might be related to combating the oxidative stress by the occurrence of active phytochemicals in it [96].

## 3. Conclusion

The fruit and fruit juice would impart beneficial effect on brain functioning. The chemical contents have properties to alleviate the harmful effect of disease by reducing the oxidation stress or by other mechanisms. The influences of dietary factors upon neuronal process and synaptic flexibility have exhibited few of the essential underlying mechanisms for the role of diet in maintaining brain health and mental activity. The more investigations are warranted to elucidate the role of phytoconstituents in therapeutics of brain disorders.

## Figures and Tables

**Table 1:** Various phytoconstituents present in fruits.

Fruits	Phytochemical compounds
Strawberries	Kaempferol, fisetin, matairesinol, secoisolariciresinol, gallic acid, ellagic acid, and chlorogenic acid
Blackberries	Ellagic acid
Blueberries	Chlorogenic acid, secoisolariciresinol, and pterostilbene
Raspberries	Ellagic Acid, quercetin, gallic acid, cyanidins, pelargonidins, catechins, kaempferol, and salicylic acid
Plum	Lutein, cyanidin
Prunes	Lutein, ursolic acid
Cherries	Limonene
Oranges	Rutin, lutein
Red grapes	Proanthocyanidins
Pomegranates	Ellagitannins, delphinidin, cyanidin, pelargonidin glycosides, catechins, gallocatechins, and prodelphinidins
